# Modulation of adipose-derived stem cell behavior by prostate pathology-associated plasma: insights from in vitro exposure

**DOI:** 10.1038/s41598-024-64625-0

**Published:** 2024-06-26

**Authors:** Sara Cruciani, Donatella Coradduzza, Francesca Balzano, Giuseppe Garroni, Emanuela Azara, Renzo Pala, Alessandro P. Delitala, Massimo Madonia, Alessandro Tedde, Giampiero Capobianco, Marco Petrillo, Cecilia Angelucci, Ciriaco Carru, Carlo Ventura, Margherita Maioli

**Affiliations:** 1 Department of Biomedical Sciences, University of Sassari, Viale San Pietro 43/B, 07100 Sassari, Italy; 2 Institute of Biomolecular Chemistry, National Research Council, 07100 Sassari, Italy; 3 Department of Medicine, Surgery and Pharmacy, University of Sassari, 07100 Sassari, Italy; 4 Department of Clinical and Experimental Medicine, Urologic Clinic, University of Sassari, Sassari, Italy; 5 Medical Oncology Unit, University Hospital (AOU) of Sassari, 07100 Sassari, Italy; 6 Laboratory of Molecular Biology and Stem Cell Engineering, Istituto Nazionale Biostrutture E Biosistemi (INBB)-Eldor Lab, Via Corticella 183, 40128 Bologna, Italy; 7 Center for Developmental Biology and Reprogramming-CEDEBIOR, Department of Biomedical Sciences, University of Sassari, Viale San Pietro 43/B, 07100 Sassari, Italy

**Keywords:** Adipose-derived stem cells, Epigenetics, Cell proliferation, Stemness genes, Stem cell niche, Conditioned media, Prostate plasma, Cell biology, Stem cells, Prostate

## Abstract

Adipose-derived stem cells (ADSCs) are promising in regenerative medicine. Their proliferation, survival and activation are influenced by specific signals within their microenvironment, also known as niche. The stem cell niche is regulated by complex interactions between multiple cell types. When transplanted in a specific area, ADSCs can secrete several immunomodulatory factors. At the same time, a tumor microenvironment can influence stem cell behavior, modulating proliferation and their ability to differentiate into a specific phenotype. Whitin this context, we exposed ADSCs to plasma samples derived from human patients diagnosed with prostate cancer (PC), or precancerous lesions (PL), or benign prostatic hyperplasia (BPH) for 4, 7 or 10 days. We then analyzed the expression of main stemness-related markers and cell-cycle regulators. We also measured cytokine production and polyamine secretion in culture medium and evaluated cell morphology and collagen production by confocal microscopy. The results obtained from this study show significant changes in the morphology of ADSCs exposed to plasma samples, especially in the presence of prostate cancer plasma, suggesting important implications in the use of ADSCs for the development of new treatments and application in regenerative medicine.

## Introduction

Stem cells are crucial in maintaining tissue homeostasis and regeneration^1^. Cell behavior is strictly related to external signals and the surrounding environment, called niche. The niche is generally shaped by stromal cells, and the growth and signaling factors they produce^2^. The organization of the niche provides anatomical and functional interactions that contribute to the maintenance of stemness and modulate the final fate of these cells^3^. These interactions are dynamic and stem cells themselves can influence or reprogram their niche^4^. Whitin the adipose tissue, adipose-derived stem cells (ADSCs) can receive several signals from the vascular stromal fraction (SVF) in which they reside, differentiating into mature adipocytes^5^. ADSCs secrete growth factors and pro-inflammatory cytokines that lead to several metabolic complications when tissue physiology is compromised^5^. Adipose tissue plays a role also in crosstalk with other tissues and organs, promoting blood vessel growth and interactions with the immune and nervous systems^6^. In fact, dysfunction in the endocrine regulation of adipose tissue is often related to the onset and development of numerous diseases^7^. The application of adult stem cells for cell‐based tissue engineering, regenerative medicine and autologous transplantations represent a promising approach for the repair of muscle, nerves, cartilage and skin^8^. Moreover, ADSCs are used for a variety of applications in plastic surgery, as post-oncologic breast reconstruction^9^. The microenvironment emerges as a central regulator influencing the destiny of a tumor, and recent advancements in research have significantly enhanced our overall comprehension of the intricate dynamics of cancer cell-stromal interactions in tumor biology^10,11^. Cancer cells coexist and evolve alongside resident cells within the tumor microenvironment. Scientific evidence unequivocally highlights the pivotal role of these cells in both the initiation and progression of tumors^12^. Noteworthy cell types in this context include cancer-associated fibroblasts, contributing to the construction of the extracellular matrix, as well as endothelial cells and pericytes responsible for the formation and function of blood vessels^13^. Immune cells also play a pivotal role, acting either to eliminate or support the tumor^14^. Additionally, the presence of stem cells capable of differentiation within or recruited to the tumor area further contributes to this complexity^15^. The tumor microenvironment (TME) plays a key role in tumor initiation and progression, and one way to understand these interactions is to analyze cellular circuits, including autocrine and paracrine reciprocal signaling^16,17^. Adipose-derived stem cells (ADSCs) are increasingly recognized in prostate cancer research due to their potential key role in the TME^18^ and thus influencing disease progression. ADSCs, abundant in adipose tissue, can differentiate into various cell types, including those comprising the TME. Moreover they also secrete cytokines regulating tumor cell proliferation and apoptosis^19,20^. These cytokines, along with chemokines, play a crucial role in modulating tumor cell behavior and immune response, contributing to the complexity of the TME^21^. The expression of these molecules increases concomitantly with tumor progression and contributes to the promotion of metastasis^22^. The clinical significance of ADSCs in prostate cancer research lies in their ability to modulate the TME and thus influence the viability of tumor cells^23–25^. However, the role of ADSCs in prostate cancer is complex, with opposite evidence suggesting both promotional and inhibitory effects on tumor growth^26–28^. These discrepancies may be attributed to variations in experimental models and features of ADSCs across different tumor types.

In conclusion, ADSCs represent a crucial cue in understanding the TME in prostate cancer thus representing a promising therapeutic target^29^. Further studies are needed to fully delineate their role and unravel their therapeutic potential^30^. In this context, polyamines, whose in vivo biosynthesis begins with intake of amino acids (arginine, lysine and methionine) with food, could also provide insights into the influence of the TME on cells, including ADSCs^31^. Polyamines are involved in various biochemical roles, including the synthesis, functioning, maintenance, and stability of nucleic acids and proteins. They also play a pivotal role in cell signaling, DNA binding, transcription, RNA splicing, and the functioning of cytoskeleton^32,33^. Moreover, genomic studies have revealed that polyamines regulate cellular metabolic pathways, facilitating the formation of subcellular compartments of cytoplasm, mitochondria, and the nucleus^34^. In the context of the TME, the metabolism of polyamines plays a key role in the regulation of normal and cancer stem cell self-renewal^35^. Polyamines can regulate the eukaryotic translation initiation factor 5A (eIF-5A), which uses spermidine as a substrate, thus affecting specific aspects of tumorigenesis^25,36^. Furthermore, the measurement of polyamines could be valuable to understand the influence of the TME on ADSCs, their stem cell potential, their anticancer activity, and their immunonutritional effects, playing a key role in the regulation of self-renewal of normal and cancer stem cells^37^.

Whitin this context, we have previously highlighted the relevance of the tumor microenvironment in stem cell dynamics, showing that an exhausted medium from human hepatocarcinoma cells (HepG2), or breast cancer cells (MCF-7), was able to remarkably affect both the stemness and proliferation of mesenchymal stem cells^38,39^. Furthermore, ADSC transplantation in a pathological environment may transform them into a highly proliferative and dangerous phenotype^39^. Starting from these previous observations, in the present paper we aimed at evaluating ADSC behavior when exposed to plasma of patients diagnosed with benign prostatic hypertrophy, precancerous prostatic lesions, and prostate cancer, in the attempt to investigate how tumor microenvironment can affect their proliferation and cell-cell interactions. Understanding the dynamics of the TME and the role of polyamines in cellular functions could provide valuable insights into the behavior of cells within the TME, including ADSCs.

## Methods

### ADSC isolation and culturing

Adipose-derived stem cells (ADSCs) were isolated from abdominal subcutaneous adipose tissue of healthy men and women (n = 6, age = 45 ± 15 years, BMI: 22 ± 3 kg/m^2^) undergoing plastic surgery, after acceptance and signing of informed consent. The study was approved by the Review Board of the Area Vasta Emilia Centro Ethics Committee (CE-AVEC). Adipose tissue was washed in PBS (Euroclone, Milan, Italy) and digested by Collagenase type I solution (Gibco Life Technologies, Grand Island, NY, USA) for 1 h at 37 °C. Cells were then washed and resuspended in a basic culture medium composed of Dulbecco’s modified Eagle’s Medium (DMEM) (Life Technologies Grand Island, NY, USA) supplemented with 20% fetal bovine serum (FBS) (Life Technologies, Grand Island, NY, USA), 200 mM L-glutamine (Euroclone, Milan, Italy), and 200 U/mL penicillin 0. 1 mg/mL streptomycin (Euroclone, Milan, Italy)^40,41^. After reaching the confluence, cells were immunomagnetically separated and characterized by flow cytometry as previously described^1^. The culture medium was changed every 3 days. ADSCs at passage 5 were then exposed for 4, 7 or 10 days to plasma of patients diagnosed with prostate cancer (PC), or precancerous lesions (PL), or benign prostatic hyperplasia (BPH). Cells used as untreated controls were maintained in the basic growing medium (Ctrl). The other cells were then divided into three groups, each exposed to a pool of plasma derived from patients specifically belonging to the different groups for the indicated durations. A group of cells was cultured in the presence of plasma from PC patients (PC); a group of cells was cultured in cultured in the presence of plasma from PL patients (PL), and a last group of cells was cultured in the presence of plasma from BPH patients (BPH).

### Gene expression analysis

Total RNA was extracted using the ChargeSwitch kit (Thermo Fisher Scientific, Grand Island, NY, USA) after 4, 7, and 10 d of culturing under the above-described conditions. Approximately 1 µg of total RNA of each sample was quantified by the NanoDrop One/OneC Microvolume UV-Vis spectrophotometer (Thermo Fisher Scientific, Grand Island, NY, USA) and then reverse transcribed using SuperScript VILO cDNA Synthesis Kit (Thermo Fisher Scientific, Grand Island, NY, USA). KAPA SYBR FAST (Sigma-Aldrich, Munich, Germany) was used for Real-time quantitative PCR in a CFX Thermal Cycler (Bio-Rad, Hercules, CA, USA). A total of 40 amplification cycles were set at 95 °C for 3 min and then cycled at 95 °C for 3 s 60 °C for 20 s. Target Ct values of each sample were normalized to a reference gene, hGAPDH. The relative values of the all genes analyzed were expressed as fold of change (2^−∆∆Ct^) of mRNA levels observed in undifferentiated ADSCs, used as control cells. The primers used (Thermo Fisher Scientific, Grand Island, NY, USA), are described in Table 1.

**Table 1:** Primer sequences.

Primer name	Forward	Reverse
hGAPDH	GAGTCAACGGAATTTGGTCGT	GACAAGCTTCCCGTTCTCAG
Oct-4	GAGGAGTCCCAGGCAATCAA	CATCGGCCTGTGTATATCCC
Sox2	CCGTTCATGTAGGTCTCGGAGCTG	CAACGGCAGCTACAGCTAGATGC
NANOG	CATGAGTGTGGATCCAGCT	CCTGAATAAGCAGATCCAT
p16INK4	CAACGCACCGCCTAGTTACGG	AACTTCGTCCTCCAGAGTCGC
p19ARF	GCCTTCGGCTGACTGGCTGG	TCGTCCTCCAGAGTCGCCCG
p21	CAAAGGCCCGCTCTACATCTT	AGGAACCTCTCATTCACCCGA
p53	TGGCCTTGAAACCACCTTTT	AACTACCAACCCACCAGCCAA

### miRNA expression

TaqMan MicroRNA Reverse Transcription Kit (Thermo Fisher Scientific, Grand Island, NY, USA) followed by polymerase chain reaction (RT-PCR) was used to evaluate the level of expression of hsa-miR-145-5p (miR-145), hsa-miR-148a-3p (miR-148) and hsa-miR-185-3p (miR-185). RNA extraction was performed using Mirvana MIRNA ISO Kit 10-40ISO (Thermo Fisher Scientific, Grand Island, NY, USA) according to manufacturer’s instructions. The Ct values for each miRNA were normalized to U6snRNA. miRNA sequences are described in Table 2.

**Table 2:** miRNA accession numbers, symbols, and sequences.

Accession ID number	Symbol	Sequence
IMAT0000437	hsa-miR-145-5p	GUCCAGUUUUCCCAGGAAUCCCU
MIMAT0000243	hsa-miR-148a-3p	UCAGUGCACUACAGAACUUUGU
MIMAT0004611	hsa-miR-185-3p	AGGGGCUGGCUUUCCUCUGGUC

### Confocal microscopy

Immunostaining was performed after 4, 7 and 10 d of culture in the above-described conditions. Cells were fixed with 4% paraformaldehyde (Sigma Aldrich Chemie GmbH, Germany) for 30 min at RT and permeabilized with 0.1% Triton X-100 (Thermo Fisher Scientific, Grand Island, NY, USA)-PBS for 1 h at RT in agitation. Cells were then washed three times in PBS and incubated with 3% bovine serum albumin (BSA)-0. 1% Triton X-100 in PBS (Thermo Fisher Scientific, Grand Island, NY, USA) for 1 h in agitation at RT.

Primary anti-rabbit anti-collagen I antibody (Abcam, United Kingdom) was incubated overnight at 4 °C in agitation. Cells were then washed twice in PBS for 5 min and incubated with fluorescence-conjugated secondary antibody (Life Technologies, USA) at 37 °C for 1 h in the dark. Nuclei were labelled with 1 µg/mL 4,6-diamidino-2-phenylindole (DAPI) (Thermo Fisher Scientific, Grand Island, NY, USA). Images are acquired under a confocal microscope (TCS SP5, Leica, Nussloch, Germany).

### ELISA assay

ADSC culture supernatants were collected after 4, 7 and 10 d of culturing in the above-described conditions. The concentrations of IL-6 and TNF-α were determined using streptavidin-HRP conjugated systems Human IL-6 Mini TMB ELISA Development kit (PeproTech EC, Ltd., London, UK) and Human TNF-α Mini TMB ELISA Development kit (PeproTech EC, Ltd., London, UK), respectively. Standard curves were prepared accordingly to manufacturer’s instructions. Each sample was assayed in duplicate, and values were expressed as the mean ± SD of 2 measures per sample.

### Polyamine detection

Liquid chromatography/ high-resolution mass spectrometry (LC-HRMS) analysis was performed using a UPLC Ultimate 3000 (Thermo Fisher-Dionex San Jose, CA, USA) system equipped with a HESI-II electrospray source to a Q-exactive orbitrap-based mass spectrometer (all from Thermo Scientific, San Jose, CA, USA). Chromatographic separation was performed on Phenomenex Gemini C18 (100 × 2 mm), 3 µm particle size, the column was held at 37 °C. Peaks were obtained at a flow rate of 0.4 mL min−1 with a sample injection volume of 5 µL^42,43^.

Q-Orbitrap HRMS (Thermo Scientific, San Jose, CA) with HESI-II electrospray source was operated in positive mode. The Xcalibur 3.1.66 software (Thermo Scientific, Bremen, Germany) was used to control the instruments and to process the data. The reference standards of putrescine, spermidine hydrochloride, spermine, agmatine sulfate salt, N-acetyl-putrescine hydrochloride, N-acetyl-spermine trihydrochloride, N-acetyl-spermidine dihydrochloride, deuterated histamine, heptafluorobutyric acid (HFBA), and methanol LC/MS grade were purchased from Sigma-Aldrich (St. Louis, MO, USA). Water for LCMS was purchased from Fisher Scientific (Fair Lawn, NJ, USA).

### Statistical analysis

For this study, Kruskal–Wallis rank sum, two-way analysis-of-variance ANOVA tests with Tukey’s correction and Wilcoxon signed-rank test were used, assuming a p value < 0.05 as statistically significant. We considered *p < 0.05, **p < 0.01, ***p < 0.001, ****p ≤ 0.0001. Statistical analysis was performed using GraphPad Prism 9.0 software (GraphPad, San Diego, CA, USA). The experiments were performed two times with three technical replicates for each treatment.

### Ethics approval and consent to participate

The study was conducted in accordance with the Declaration of Helsinki and approved by the Area Vasta Emilia Centro Ethics Committee (CE-AVEC) n_EM468-2019 6/2016/U/Tess/AOUBo 22 May 2019, Ethical committee, Policlinico Sant’Orsola AOU Bologna. Informed consent was obtained from all individual participants included in the study.

## Results

### Morphological analysis of ADSCs exposed to plasma samples

**Figure 1:**
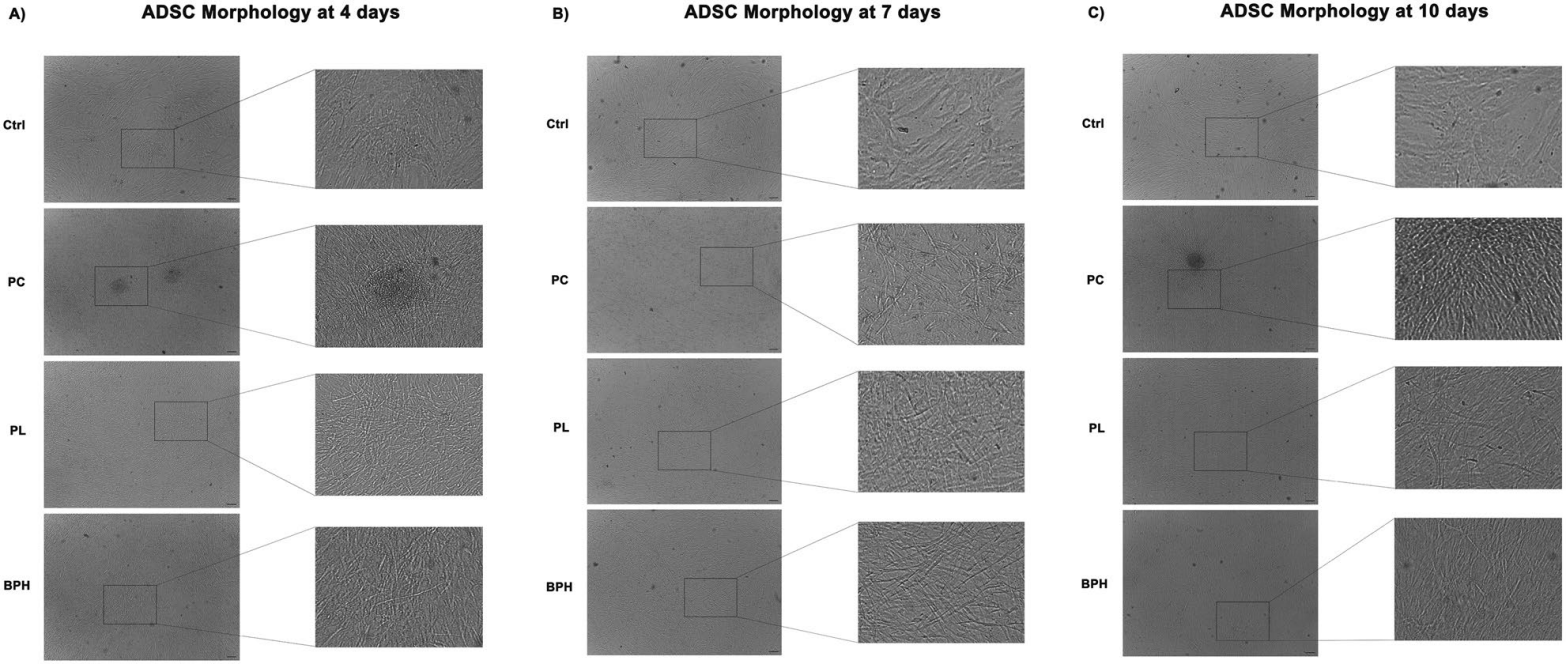
Optical microscope analysis of ADSC morphology after exposure to PC, PL and BPH plasma samples. Figure shows morphological changes in cell exposed to plasma after 4 (**A**), 7 (**B**) and 10d (**C**), as compared to untreated control cells (Ctrl). Scale bar = 100 µm.

ADSC morphology was evaluated under an optical microscopy (Leica, Nussloch, Germany) after 4 (Panel A), 7 (Panel B) and 10 (Panel C) days in culture in the presence of different plasma samples (Fig. 1). We observed significant changes in morphology of ADSCs exposed to plasma samples, as compared to control untreated cells (Ctrl). For all conditions, but especially in the presence of PC plasma, cells appeared extremely more confluent, even growing over multiple layers, than controls cells, for each time point analyzed. These data were further confirmed by the BrdU Cell Proliferaion assay and B-galactosidase colorimetric staining (Supplementary information).

### Exposure of ADSCs to plasma samples increases stemness-related genes

**Figure 2:**
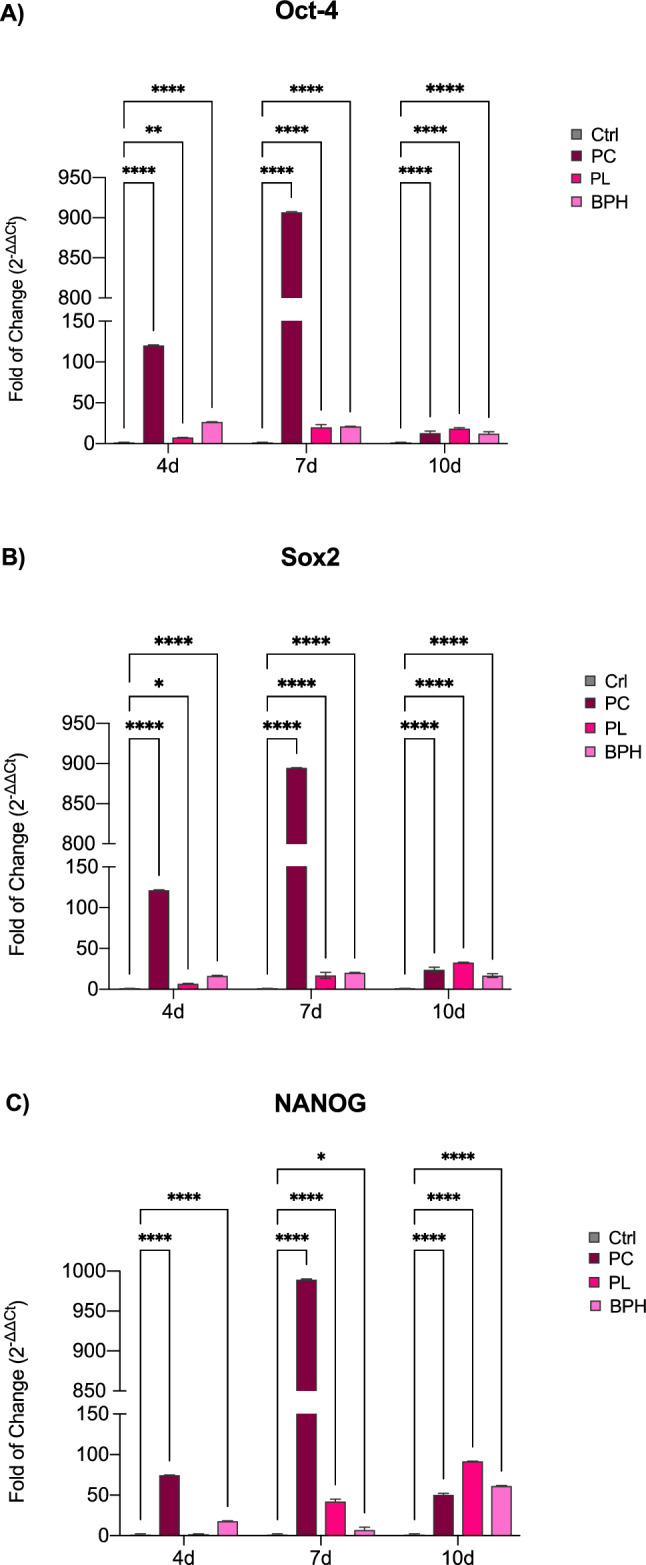
Expression of stemness genes. The expression of stemness-related genes Oct-4 (**A**), Sox2 (**B**) and NANOG (**C**) was evaluated in ADSCs exposed to PC, PL or BPH plasma samples after 4, 7 and 10d in culture. The mRNA levels for each gene were normalized to Glyceraldehyde-3-Phosphate-Dehydrogenase (GAPDH) and expressed as fold of change (2^−ΔΔCt^) of the mRNA levels observed in untreated control cells (Ctrl) defined as 1 (mean ± SD; n = 6). Data are expressed as mean ± SD referred to the control (*p ≤ 0.05), (**p ≤ 0.01), (***p ≤ 0.001), (****p ≤ 0.0001).

Figure 2 shows the levels of expression of stemness-related genes in ADSCs exposed to PC, PL or BPH plasma samples after 4, 7 and 10d in culture. The expression of all the tested stemness genes was significantly increased in cells exposed to plasma, as compared to control untreated cells. This effect is extremely marked in PC-exposed ADSCs from the first days of treatment, reaching a pick at the end of 7 days.

### Exposure of ADSCs to plasma samples affect cell cycle-regulator genes

**Figure 3:**
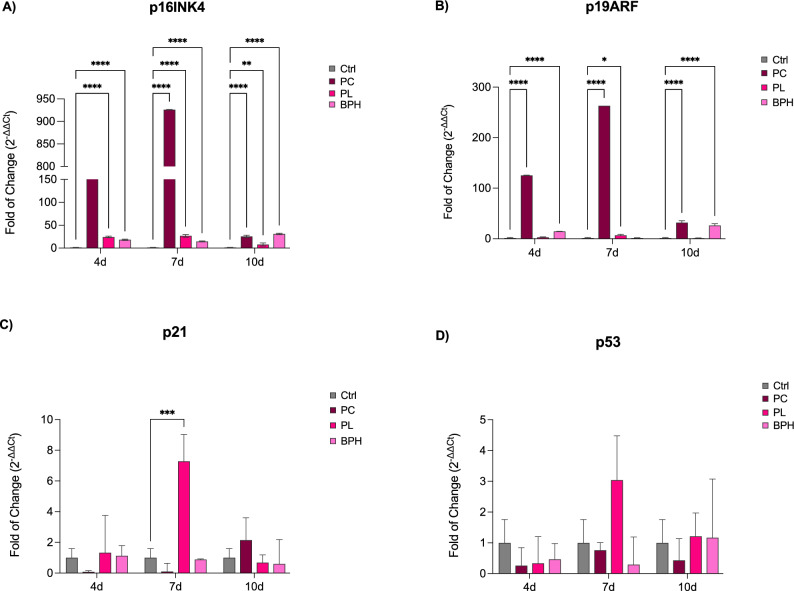
Expression of cell-cycle regulator genes. The expression of p16 (**A**), p19 (**B**) p21 (**C**) and p53 (**D**) was evaluated in ADSCs exposed to PC, PL or BPH plasma samples after 4, 7 and 10d in culture. The mRNA levels for each gene were normalized to Glyceraldehyde-3-Phosphate-Dehydrogenase (GAPDH) and expressed as fold of change (2^−ΔΔCt^) of the mRNA levels observed in untreated control cells (Ctrl) defined as 1 (mean ± SD; n = 6). Data are expressed as mean ± SD referred to the control (*p ≤ 0.05), (**p ≤ 0.01), (***p ≤ 0.001), (****p ≤ 0.0001).

Figure 3 shows the levels of expression of cell-cycle-regulatory genes in ADSCs exposed to PC, PL or BPH plasma samples after 4, 7 and 10d in culture. The expression of p16 (Panel A) and p19 (Panel B) was significantly increased in cells exposed to plasma samples, as compared to control untreated cells. This effect is extremely marked in PC-exposed ADSCs from the first days of treatment, reaching a pick at the end of 7 days. A completely different trend was observed for p21 (Panel C) and p53 (Panel D), whose expression did not show markedly different levels from controls, except for PL-exposed ADSCs, being upregulated at the end of 7 days in culture.

### Exposure of ADSCs to plasma samples infuences miRNA profiles

**Figure 4:**
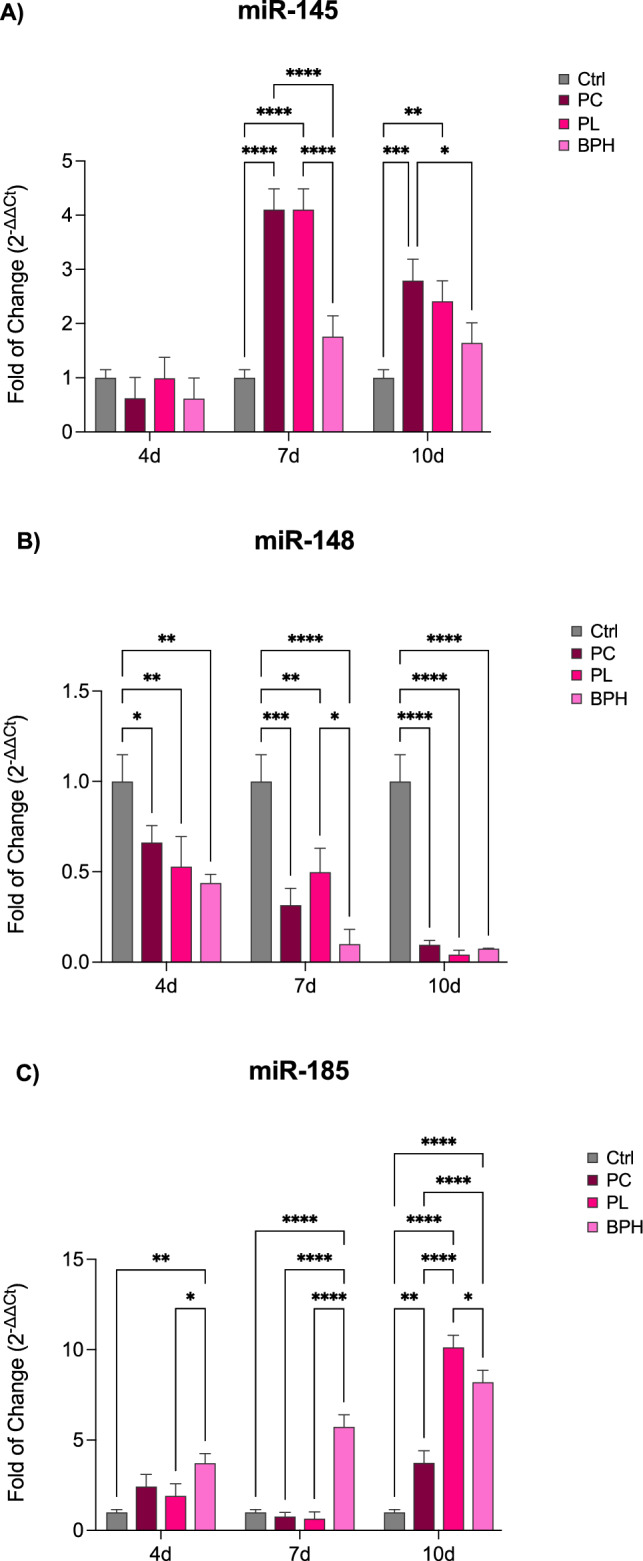
Expression of miRNAs after 4, 7 and 10 days of culturing. The expression of miR-145 (**A**), miR-148a (**B**) and miR-185 (**C**) was evaluated in ADSCs exposed to PC, PL or BPH plasma samples after 4, 7 and 10d in culture. The mRNA levels for each gene were normalized to U6snRNA and expressed as fold of change (2^−∆∆Ct^) of the mRNA levels observed in untreated control cells (Ctrl) defined as 1 (mean ± SD; n = 6). Data are expressed as mean ± SD referred to the control (*p ≤ 0.05), (**p ≤ 0.01), (***p ≤ 0.001), (****p ≤ 0.0001).

miRNAs expression was evaluated in cells exposed to plasma samples after 4, 7 and 10 days in culure. As showed in Fig. 4, miR-145 (Panel A) was significantly upregulated after 7 and 10d in culture, for all conditions, as compared to control untreated cells. An opposite trend could be observed for miR-148 expression (Panel B), being significantly downregulated in ADSCs cultured in the presence of plasma samples from PC, PL and BPH patients, as compared to control untreated cells. miR-185 (Panel C) showed a completely different trend, being significantly upregulated for cells cultured in the presence of BPH plasma samples from the first days in culture, while was significantly upregulated for PC and PL samples only at the end of 10d, as compared to control untreated cells.

### Collagen I deposition in cells exposed to plasma samples

**Figure 5:**
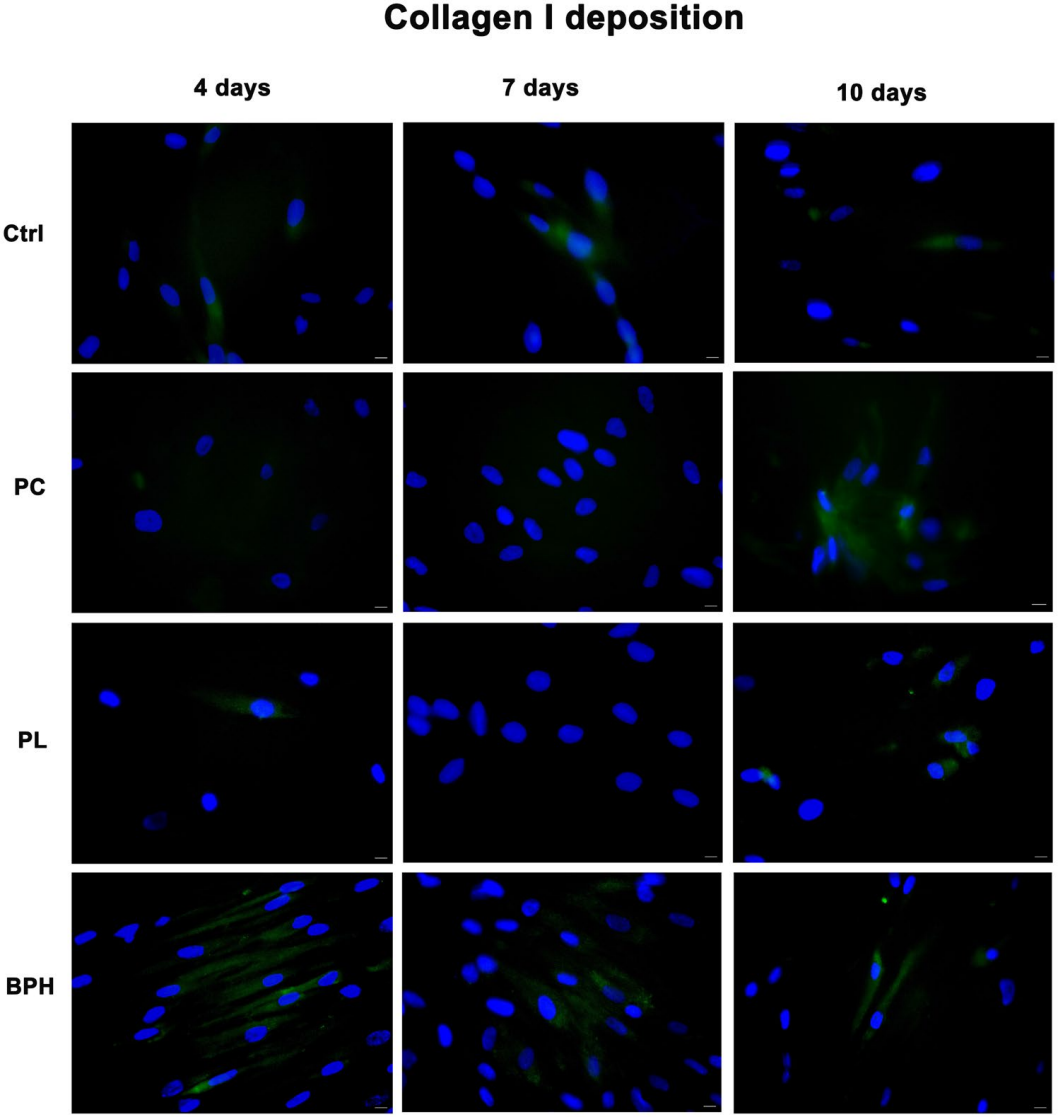
Analysis of collagen deposition. Immunohistochemical analysis of the expression of Collagen type I was assessed in ADSCs exposed to PC, PL or BPH plasma samples after 4, 7 and 10d in culture. Nuclei are labelled with 4,6-diamidino-2-phenylindole (DAPI, blue). Scale bars: 40 µm. The figures are representative of different independent experiments. Fields with the highest yield of positively stained cells are shown.

Figure 5 shows the expression of collagen I in cells exposed PC, PL or BPH plasma samples, as compared to control untreated cells. Collagen deposition was visible at the end of 10 days in ADSCs exposed to PC plasma, while was higher after 4d in cells cultured in the presence of BPH plasma. No differences were observed for PL, as compared to control untreated cells.

### Plasma samples increase the release of pro-inflammatory cytokines

**Figure 6:**
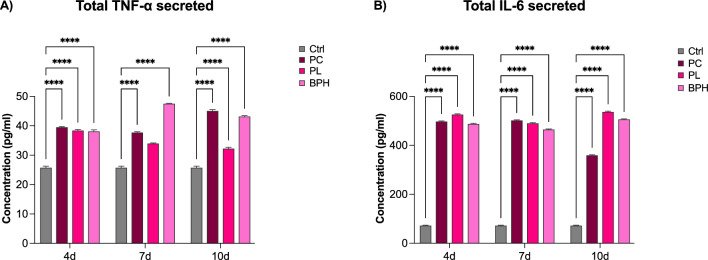
TNF-α and IL-6 quantification by ELISA. The concentration of TNF-α (**A**) and IL-6 (**B**) was measured in ADSCs exposed to PC, PL or BPH plasma samples after 4, 7 and 10d in culture, as compared to control untreated cells. Data are expressed as mean ± SD referred to the control (mean ± SD; n = 6) Data are expressed as mean ± SD referred to the control (*p ≤ 0.05), (**p ≤ 0.01), (***p ≤ 0.001), (****p ≤ 0.0001).

The secretion of proinflammatory cytokines TNF-α and IL-6 and was evaluated by ELISA in ADSCs exposed to plasma samples from PC, or Pl or BPH patients after 4, 7 and 10d in culture. The ELISA (Fig. 6) revealed significantly increased concentrations of TNF-α and IL-6 in supernatants of cells exposed to PC, PL and BPH samples for all time points analyzed. The release of TNF-α reached a maximum in BPH-treated ADSCs, as compared to control untreated cells (Panel A), while for IL-6 the higher concentrations were measured in in cells exposed to PL (Panel B), as compared to untreated control ADSCs.

### Polyamine levels

In the studies performed, over four, seven, and ten days, several polyamine precursors, as arginine and lysine, and their metabolites, were analyzed in relation to different disease categories using ANOVA, the Schefficé test, and the Kruskal–Wallis test. In the four-day study, Lysine, Acetylputrescine, Acetylspermidine, Arginine, GABA, Ornithine, Spermine, and Spermidine were found. For all the substances, the ANOVA test showed a significant difference among disease categories. The Schefficé test for all pairwise comparisons indicated that all groups were statistically different from each other. The residuals exhibited a normal distribution in all cases. For Ornithine, the Kruskal–Wallis test confirmed significant differences, and the Conover post-hoc analysis highlighted specific pairwise differences, Table 3.

**Table 3:** Polyamine concentrations on 4-days. Unit are express as ng/mL. The ‘NF’ in the table stands for ‘Not Found’.

Polyamines 4 days	Ctrl	PC	PL	BPH	Unit
Arginine	63.29	NF	0.152	0.352	ng/mL
Ornithine	26.909	24.408	20.156	25.872	ng/mL
Spermine	0.046	0.106	0.08	0.101	ng/mL
Spermidine	0.148	0.163	0.137	0.131	ng/mL
Agmatine	0.002	0.15	NF	NF	ng/mL
GABA	0.012	0.058	0.065	0.044	ng/mL
Acetylputrescine	0.036	0.054	0.069	0.058	ng/mL
Acetylspermidine	0.003	0.018	0.019	0.041	ng/mL
Acetylspermine	0.007	0.013	0.009	0.015	ng/ml
Lysine	34.334	33.214	31.776	32.17	ng/mL
Cadaverine	0.018	NF	NF	NF	ng/mL

In the seven-day study, Agmatine, Acetylputrescine, Acetylspermidine, Arginine, Cadaverine, Gaba, Lysine, Ornithine, Spermine, and Spermidine were found. For all the substances except Cadaverine, Gaba, Lysine, and Ornithine, the ANOVA test showed a highly significant difference among groups (p < 0.001). The Schefficé test for all pairwise comparisons indicated that all groups were statistically different from each other. For Cadaverine, the Kruskal–Wallis test was significant (p = 0.024914), indicating a difference among groups. The Conover post-hoc analysis identified specific pairwise differences. For Gaba, Lysine, and Ornithine, the ANOVA test showed a highly significant difference among groups (p < 0.001). The Schefficé test for all pairwise comparisons indicated that all groups were statistically different from each other. The Kruskal–Wallis test was confirmed significant, and the Conover post-hoc analysis highlighted specific pairwise differences, Table 4.

**Table 4:** Polyamine concentrations on 7-days. Unit are express as ng/mL. The ‘NF’ in the table stands for ‘Not Found’.

Polyamines 7 days	Ctrl	PC	PL	BPH	Unit
Arginine	63.29	NF	0.255	0.438	ng/mL
Ornithine	26.909	22.288	25.165	24.912	ng/mL
Spermine	0.046	0.039	0.044	0.09	ng/mL
Spermidine	0.148	0.574	0.194	72.321	ng/mL
Agmatine	0.002	NF	0.034	0.596	ng/mL
GABA	0.012	0.064	0.077	0.074	ng/mL
Acetylputrescine	0.036	0.062	0.086	0.378	ng/mL
Acetylspermidine	0.003	0.012	0.017	0.027	ng/mL
Acetylspermine	0.007	NF	NF	0.012	ng/ml
Lysine	34.334	29.907	36.887	20.386	ng/mL
Cadaverine	0.018	0.016	4.563	24.687	ng/mL

In the ten-day study, Acetylputrescine, Acetylspermidine, Acetylspermine, Agmatine, Cadaverine, Gaba, Lysine, Ornithine, Spermidine, and Spermine were found. For all the substances except Spermine, the ANOVA test showed a significant difference between disease groups (p < 0.001). The Schefficé test for all pairwise comparisons indicated that all groups were statistically different from each other. The D’Agostino-Pearson test for Normal distribution confirmed the normal distribution of residuals in all cases except for Arginine, where the normality assumption was violated (p = 0.0166). For Spermine, the Kruskal–Wallis test was significant (p = 0.024914), indicating a difference in the medians of the groups. The Conover post-hoc analysis identified specific pairwise differences. The Arginine concentration analysis showed consistent concentration with minimal variation in the lowest disease level (Factor 2). However, the Kruskal–Wallis test was significant (p = 0.024914), indicating a difference in Arginine levels between disease groups. The Conover post-hoc analysis identified specific pairwise differences, Table 5. In conclusion, this comprehensive analysis of biochemical markers reveals significant variations in molecular profiles associated with different disease categories.

**Table 5:** Polyamine concentrations on 10 days. Unit are express as ng/mL. The ‘NF’ in the table stands for ‘Not Found’.

Polyamines 10 days	Ctrl	PC	PL	BPH	Unit
Arginine	63.29	0.144	0.227	0.354	ng/mL
Ornithine	26.909	46.044	28.329	19.455	ng/mL
Spermine	0.046	0.083	0.037	0.049	ng/mL
Spermidine	0.148	1.24	0.104	25.964	ng/mL
Agmatine	0.002	0.15	0.013	0.359	ng/mL
GABA	0.012	0.07	0.083	0.085	ng/mL
Acetylputrescine	0.036	0.314	0.086	0.49	ng/mL
Acetylspermidine	0.003	0.013	0.013	0.021	ng/mL
Acetylspermine	0.007	0.024	0.007	0.01	ng/ml
Lysine	34.334	20625	30.145	22.98	ng/mL
Cadaverine	0.018	17.925	2.314	21.918	ng/mL

## Discussion

This research explores the impact of benign prostatic hypertrophy, precancerous prostatic lesions, and prostate cancer plasma samples in adipose-derived stem cell behavior, with the aim to better understand how the tumor microenvironment might affect their proliferation and intercellular interactions. ADSCs are cells with a high degree of metabolic and interlinear plasticity, which makes them good candidates for regenerative therapeutic protocols^44^. ADSCs are known to be influenced by the environment, known as *niche*, in which they grow, and produce their signaling players. The niche establishes anatomical and functional interactions that contribute sustaining stemness and modulating the final fate of these cells^45,46^. Here, ADSCs isolated from human abdominal subcutaneous adipose tissue of healthy individuals, were exposed to plasma from patients with prostate cancer, precancerous lesions, and benign prostatic hypertrophy. In addition to visible changes in cell morphology after culturing in the presence of plasma, the gene expression analysis revealed that the main stemness-related markers Oct-4, Sox2 and NANOG were significantly upregulated when cells were exposed to PC samples, since the first days in culture, reaching a peak after 7 days. PL and BPH samples were also able to increase the expression of these markers, but with a more pronounced upregulation at the end of 10 days. This effect is in line with what it has been previously observed by us and other authors, demonstrating that exhausted MCF7 medium increases stem cell potency of ADSCs^39,47^, playing an important role in transforming cells in a malignant and aggressive phenotype. Epigenetics is also involved in regulating cell differentiation and development^48^. Differential expression of microRNAs is a critical epigenetic mechanism involved in the development and progression of human PC^49^. We previously demonstrated that miR-145, miR-148, and miR-185 were differentially expressed among patients with PC, PL and BPH^26^. Here, we analyzed the expression of the same markers in ADSCs exposed to plasma samples. miR-145 was significantly upregulated after 7 and 10d in culture especially after treatment with PC and PL samples. MiR-145 overexpression promotes ADSC proliferation and migration^50^. Increased levels of this miRNA in our cells seems to be related to increased levels of stemness-associated markers, maintaining their proliferating and undifferentiating capabilities in tumor microenvironment. MiR-148b-3p is suggested as an indicator to distinguish malignant from benign prostate disease, being downregulated in BPH as compared to PC^51^. Intriguingly, we observed a significant downregulation of miR-148 in ADSCs exposed to BPH plasma at a level below that detected in control cells. This finding is particularly rewarding in light of the epigenetic role of miRNAs^36^. Moreover, inhibition ofmiR-148 has been shown to result in neural stem cell differentiation and recovery from ischemic stroke in rats^37^, suggesting that the currently observed downregulation of miR-148 by BPH plasma may entail a protective effect on ADSC homeostasis. MiR-185 also plays important roles in numerous cancer types. It has been shown to be a potential prognostic biomarker for early-stage hepatocellular carcinoma^52^. miR-185-3p also inhibits the invasion and metastasis^53^. To this end, the upregulation of miR-185 in both BPH-, and PL-exposed ADSCs may represent a mechanism counteracting a stem cell drift towards uncontrolled growth regulatory dynamics. On the contrary, its lower expression in PC-, as compared to BPH- and PL-exposed ADSCs may reflect a different adaptive stem cell response in a milieu signaling a disease progression towards malignancy. All these significant modifications in the expression of key genes controlling cell growth and proliferation are directly related to the morphological changes observed in treated cells. Tumor and tumor-associated stromal cells promote the production and remodeling of extracellular matrix. Among the major matrix-forming proteins are members of the collagen family^54^. ADSCs are involved in ECM production, by secretion of collagen and fibronectin^55^. Here, we observed that type I collagen deposition was increased after treatment with BPH plasma, as compared to control cells samples, while being undetectable in PC or PL-treated cells. This suggests that ADSCs may have better retained the capability for structuring their architecture and tissue repair potential when exposed to a “benign” microenvironment, than in the presence of a medium enriched in pre-neoplastic or malignant signaling. In addition, the ELISA assay was used to measure the concentrations of IL-6 and TNF-α, which are critical in the regulation of the inflammatory response in the tumor microenvironment. Both factors are involved in tumor growth and metastasis, and changes in their concentrations appear to be directly related to the extent of disease^56,57^. Elevated IL-6 levels have been associated with advanced stage and metastasis-related morbidity^58^. IL-6 also plays an important role in the maintenance of stem/progenitor cells, diffusing through the cellular structures and tissues of the tumor microenvironment due to its low molecular weight^59,60^. ADSCs exposed to plasma samples show elevated levels of both proinflammatory cytokines, especially for PC and BPH. Other authors analyzed serum levels of IL-6 and TNF-α in subjects with PC and those with BPH in different age groups. Subjects with PC had higher levels of both variables than subjects with BPH. These increased levels seem to support the hypothesis that inflammation may play a significant role in the pathogenesis of PC^57,61^. Moreover, among the various forms of cancer including breast cancer, inflammation associated with the tumor microenvironment contributes to each stage of cancer progression and induces a self-reinforced senescence/inflammatory environment, which is responsible for epithelial plasticity and stemness characteristics that tend toward a more aggressive phenotype^62–64^. Cellular senescence blocks tumorigenesis by inducing cell cycle arrest in damaged and mutated cells. However, senescent stromal cells often release paracrine signals that can promote tumorigenesis, conferring therapeutic resistance in advanced cancers^65,66^. In this context, exposure of cells especially to the plasma of subjects with PC, seems to induce the expression of key senescence-associated markers, particularly p16 and p19, contributing to the maintenance of this malignant phenotype. In contrast, p21 and p53, key factors in the regulation of cell growth^67^, are induced following exposure to PL. Activation of these genes in ADSCs would apparently counteract progression to a malignant phenotype and suppress tumor growth. Finally, polyamine detection was used to understand the influence of the tumor microenvironment on ADSCs, their stem cell potential, their anticancer activity, and their immunomodulating effects. The studies conducted over four, seven, and ten days reveal significant variations in molecular profiles associated with different disease categories. This includes the analysis of several substances, such as arginine and lysine and their metabolites, polyamines. The ANOVA test showed a significant difference among disease categories for all substances, with the Schefficé test confirming statistical differences among all groups. In the four-day study, the residuals exhibited a normal distribution in all cases, and for Ornithine, the Kruskal–Wallis test confirmed significant differences, with the Conover post-hoc analysis identifying specific pairwise differences. In the seven-day study, the ANOVA test showed a highly significant difference among groups for all substances except Cadaverine, Gaba, Lysine, and Ornithine. For Cadaverine, the Kruskal–Wallis test was significant, indicating a difference among groups. The Conover post-hoc analysis identified specific pairwise differences. In the ten-day study, the ANOVA test showed a significant difference between disease groups for all substances except Spermine. For Spermine, the Kruskal–Wallis test was significant, indicating a difference in the medians of the groups. The Conover post-hoc analysis identified specific pairwise differences. In conclusion, the comprehensive analysis of biochemical markers revealed significant variations in molecular profiles associated with different disease categories. Identified markers hold promise for further exploration in clinical diagnostics and therapeutic interventions, contributing to enhanced health assessment.

## Conclusions

The results obtained from this study show significant changes in the morphology of ADSCs exposed to plasma samples, especially in the presence of prostate cancer plasma. The levels of expression of stemness-related genes in ADSCs exposed to PC, PL or BPH plasma samples were significantly increased after 4, 7, and 10 days in culture. This suggests that the tumor microenvironment can influence the stemness of ADSCs, promoting their proliferation. In conclusion, these findings could have significant implications for the development of treatments for these diseases, including the use of ADSCs for cell-based tissue engineering, regenerative medicine, and autologous transplantations.

### Supplementary Information

Supplementary Figures.

## Data Availability

All data supporting the findings of this study are available within the paper.
