# Supplementary material for: Modulation of adipose-derived stem cell behavior by prostate pathology-associated plasma: insights from in vitro exposure
Source: Sci Rep. 2024 Jun 26;14:14765. doi: 10.1038/s41598-024-64625-0 (PMC11208502; doi:10.1038/s41598-024-64625-0)
Supplement: Supplementary file 1 — Supplementary Figures. [file 41598_2024_64625_MOESM1_ESM.docx]

**Supplementary Information**

Supplementary Figure 1 shows proliferation of ADSCs after exposure to PC, PL or BPH plasma samples after 4, 7 and 10d in culture, as compared to control untreated cells. As observed from morphological analysis, cells exposed to plasma samples significantly increased their proliferation rate starting from the beginning. This effect was visible for all conditions analyzed at different time points.

**Figure S1.** ADSC proliferation after 4, 7 and 10d in culture in the presence of PC, PL or BPH plasma samples, as compared to control untreated cells. Cell proliferation is expressed in OD units as compared to control untreated cells. Data are expressed as mean ± SD referred to the control (* p ≤ 0.05), (** p ≤ 0.01), (*** p ≤ 0.001), (**** p ≤ 0.0001).

ADSCs cultured in the presence of plasma samples, after 4, 7 or 10 days in culture, showed a reduced number of blue-positive cells, as compared to control untreated cells, as highlight by Senescence β-Galactosidase Staining especially after 7 and 10 days in culture.


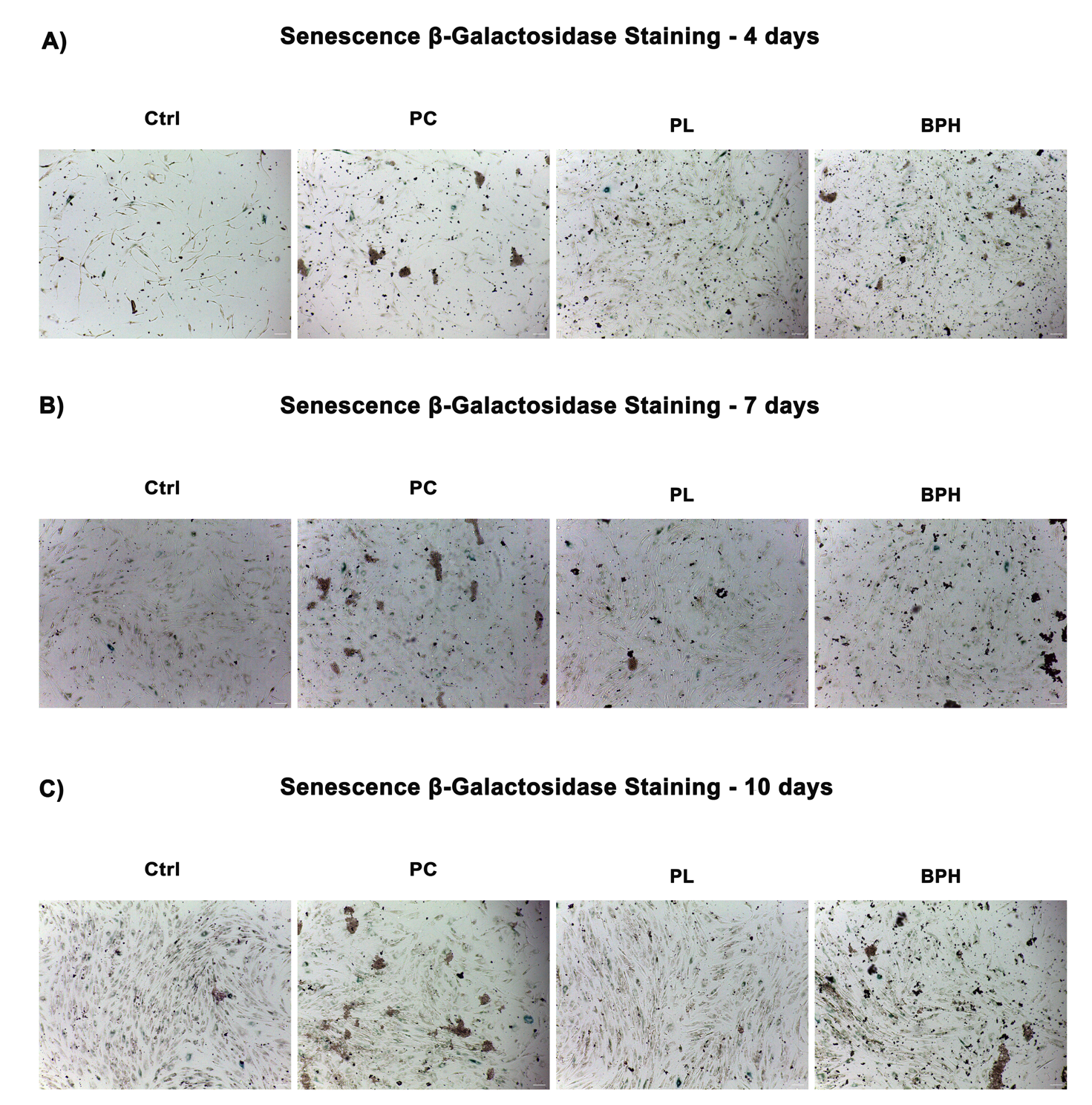


**Figure S2.** β-Galactosidase Staining of ADSC cultured in the presence of PC, PL or BPH plasma samples after 4 (Panel A), 7 (Panel B) and 10d (Panel C), as compared to control untreated cells. Scale bar=100 μm**.**

**Figure S3.** The percentage of SA-𝛽-Gal-positive cells for each treatment was calculated as the number of positive cells divided by the total number of cells counted using an image software analysis (ImageJ). Data are expressed as mean ± SD referred to the control (* p ≤ 0.05), (** p ≤ 0.01), (*** p ≤ 0.001), (**** p ≤ 0.0001).
